# Climate Warming Increases the Voltinism of Pine Caterpillar (*Dendrolimus spectabilis* Butler): Model Predictions Across Elevations and Latitudes in Shandong Province, China

**DOI:** 10.3390/insects16030249

**Published:** 2025-02-28

**Authors:** Yongbin Bao, Teri Gele, Xingpeng Liu, Zhijun Tong, Jiquan Zhang

**Affiliations:** 1 School of Environment, Northeast Normal University, Changchun 130024, China; baoyb924@nenu.edu.cn (Y.B.); tergl@nenu.edu.cn (T.G.); liuxp912@nenu.edu.cn (X.L.); gis@nenu.edu.cn (Z.T.); 2 Key Laboratory for Vegetation Ecology, Ministry of Education, Changchun 130024, China; 3 State Environmental Protection Key Laboratory of Wetland Ecology and Vegetation Restoration, Changchun 130024, China

**Keywords:** climate change, climate scenarios, voltinism, pine caterpillar, response

## Abstract

The pine caterpillar (*Dendrolimus spectabilis* Bulter, Lepidoptera: Lasiocampidae) is a destructive insect threatening forest communities across Eurasia. The pest is polyvoltine, and under global warming, more favorable temperatures can lead to additional generations. Here, we simulated the pine caterpillar voltinism under current and future climatic scenarios based on insect thermal physiology and cumulative growing degree day (CGDD) model. Subsequently, we revealed the future change patterns of the voltinism along elevational and latitudinal gradients. The results showed that both CGDD and pine caterpillar voltinism are increasing. The current voltinism of pine caterpillar ranges from 1.26 to 1.56 generations (1.40 ± 0.07), with an increasing trend of 0.04/10a. Similar trends are expected to continue under the future climate scenarios, with values of 0.01/10a, 0.05/10a, 0.07/10a, and 0.09/10a for the SSP1-2.6, SSP2-4.5, SSP3-7.0, and SSP5-8.5 scenarios, respectively. At the elevation and latitudinal gradients, voltinism increases across all ranges, peaking at 500–1000 m and latitudes of 34–34.5° N. This study highlights that the increase in voltinism is not limited to low-elevation and -latitude regions but is predicted across various elevations and latitudes. These findings can enhance our understanding of how climate change affects pine caterpillar voltinism and contribute to forest pest management strategies, although this study assumes a linear relationship between temperature and voltinism, without considering other ecological factors.

## Simple Summary

The pine caterpillar (*Dendrolimus spectabilis* Butler) is a major pest that damages coniferous forests in Shandong Province, China. With global warming, insect outbreak is expected to pose a more frequent threat to forest communities. However, the extent to which rising temperatures affect pine caterpillar populations, particularly across different elevations and latitudes, has not been well understood. Our study found that warmer temperatures lead to an increase in the number of generations (voltinism) of pine caterpillars, with one to two additional generations predicted. Furthermore, the distribution patterns of pine caterpillars across elevations and latitudes are increasing over time. These findings can provide insights for understanding the effects of climate change on forest insect pests.

## 1. Introduction

Climate change has been associated with an increase in insect pest pressures worldwide [1,2,3,4]. This pressure includes changes in development rate, overwintering survival rates, life history, voltinism, and population dynamics and geographical distribution [3,5,6,7]. It is anticipated that there can be an increase in the frequency and intensity of insect outbreaks, posing a threat to ecosystems [8]. Therefore, insect pest species are considered a significant challenge under the impact of climate change.

Research has indicated that increasing temperature represents the primary climate change factor that directly affects herbivores [9,10]. Milder winters increase the survival rates of insects during winter months [11,12,13]. Warmer springs can lead to earlier larval hatching, the earlier onset of the flight period, and increased capacity for multiple generations of species per year [14,15,16]. However, from an ecological and evolutionary perspective, voltinism may prove to be a more significant factor than seasonality, as it allows for the acceleration of population growth or adaptation across more generations [17]. A reasonable explanation is that rising temperatures can expedite development rates and enhance metabolism, leading to an increase in the number of generations of polyvoltine insects and greater food intake, which may result in more severe damage to host plants [9]. It is expected that in 2050 and 2070, regions with an increase in the number of generations may create more favorable conditions for the population growth of *Spodoptera eridania* (Lepidoptera: Noctuidae), thereby amplifying the probability of its outbreak [18]. Therefore, a deeper understanding of the relationship between climate and insect pests, particularly through the parameter of voltinism, is essential for assessing the impact of climate change on insects.

With reference to the previous research, the present study defines the voltinism as “the number of generations in a year” [18,19]. Ectotherms benefit from temperature during their developmental stages, with polyvoltine insects being particularly sensitive to temperature variations [20,21,22]. Therefore, the relationship between temperature and insect development is often used to establish predictive models, including both linear and nonlinear approaches, to understand insect responses to temperature changes [21,23,24,25]. Among these, the growing degree day (GDD) method is widely adopted due to its simplicity, ease of use, and high interpretability [18,20]. This method enables researchers to intuitively quantify the impact of temperature on the life history of various insect species. However, its primary limitation lies in the assumption of a linear relationship between temperature and developmental rate, which may fail to accurately represent complex biological responses, especially under extreme temperature conditions. In contrast, nonlinear models offer greater flexibility in capturing the effects of temperature changes on development but often require more data and complex parameter estimations, increasing the risk of overfitting. This study employs the GDD method as the main analytical tool due to its intuitiveness and relative simplicity in predicting insect development, making it suitable for a preliminary assessment of climate change impacts. Most studies that use this model to estimate voltinism predict that the number of generations is expected to increase under future climate scenarios [18,26,27,28,29]. However, as global warming is unevenly distributed across different regions, estimates of this variability may depend on geographical location [30,31].

Simultaneously, the response of insect populations to temperature exhibits geographic diversity, with greater sensitivity demonstrated in regions characterized by shorter growing seasons at northern latitudes [32]. It is anticipated that future temperature conditions will promote an increase in insects’ voltinism per year in low-elevation areas and enable them to migrate to higher areas [7]. The outbreaks of some defoliators are also related to elevation [33,34]. There is some evidence that recent temperature increases have led to prolonged insect outbreaks, such as those of spruce budworm, larch budmoth, and bark beetles, at higher latitudes and elevations than those in the past [35,36,37,38].

The pine caterpillar, *Dendrolimus spectabilis* Bulter (Lepidoptera: Lasiocampidae), is a defoliating insect that feeds mainly on the pine trees. Their outbreak can result in the shedding of needles and tree mortality, even threatening forest ecosystems [39,40]. As an ectothermic and polyvoltine insect, the pine caterpillar is highly sensitive to temperature, making it an excellent model for temperature-based research [14,16,41,42]. However, the development and survival of pine caterpillars also have existing temperature limits under natural conditions [43]. Extreme temperatures, such as those during cold winters or dry summer periods, can induce diapause or even cause mortality. In temperate regions, low temperature is the dominant factor inducing diapause [25]. Research has shown that *Dendrolimus spectabilis* typically enters diapause in October, coinciding with the onset of overwintering [44]. This phenomenon is closely linked to the fixed life-history strategy developed by pine caterpillars through long-term evolution. Specifically, larvae in the third to fifth instar stages enter diapause as an adaptation to low-temperature conditions [44,45]. In contrast, there is currently no conclusive evidence suggesting that *Dendrolimus spectabilis* exhibits summer diapause in response to high temperature in China. However, experimental studies on the congeneric species *Dendrolimus punctatus* have demonstrated that exposure to daily maximum temperatures exceeding 36 °C for 26 consecutive days reduces the population density of first- to third-instar larvae [46]. Meanwhile, fourth-instar and older larvae exhibit enhanced thermal tolerance and do not display diapause behavior under the same conditions. One study further concluded that higher mean daily temperature generally accelerates the developmental rate of *Dendrolimus punctatus*. For instance, an increase of 3 °C in the mean daily temperature can advance larval development by approximately 4 days [46]. This indicates that a rising mean temperature, whether constant or fluctuating, can shorten the developmental duration of pine caterpillars. Although high temperature may occasionally induce diapause under specific conditions (e.g., summer diapause triggered by heat and drought), low temperature remains the predominant factor promoting the initiation and maintenance of diapause, typically in conjunction with short photoperiods. Numata and Shintani (2023) showed that climate warming increases the effective cumulative temperature and developmental rate of most insects [47]. Under favorable environmental conditions, such diapause could evolve into producing more generations per year [48,49], as reported in species like *Ips typographus* (Coleoptera: Scolytinae) [50], *Phratora vulgatissima* (Coleoptera: Chrysomelidae) [51], and *Dendrolimus spectabilis* [52,53]. Since the photoperiodic induction of diapause in these insects is primarily temperature-dependent, higher temperatures generally suppress diapause induction [47]. Nevertheless, changes in *Dendrolimus spectabilis* Bulter development remain largely unexplored, particularly with regard to rising temperature. While it is predicted that climate warming may lead to an increase in voltinism in some Lepidoptera species, it is still unclear whether such changes have occurred. The lack of long-term data makes this issue more indistinct, especially considering the extent of climate change in recent decades and the importance of voltinism in ecology and evolution. Most studies are combined with future climate data developed by the Intergovernmental Panel on Climate Change (IPCC) [10,54,55,56]. The Coupled Model Comparison Project Phase 6 (CMIP6) provides an effective tool to predict the future climate changes [57].

Hence, we explored the potential impact of climate warming on pine caterpillar voltinism based on insect thermal physiology and the growing degree day model. Specifically, this study aimed to (1) analyze the changes in the cumulative growing degree day under current and future climate scenarios; (2) predict the current and future variations in pine caterpillar voltinism; and (3) reveal the future change patterns of pine caterpillar voltinism across elevation and latitudinal gradients. This study provides a reference for understanding the response of pine caterpillars to climate change.

## 2. Materials and Methods

### 2.1. Study Area

**Figure 1:**
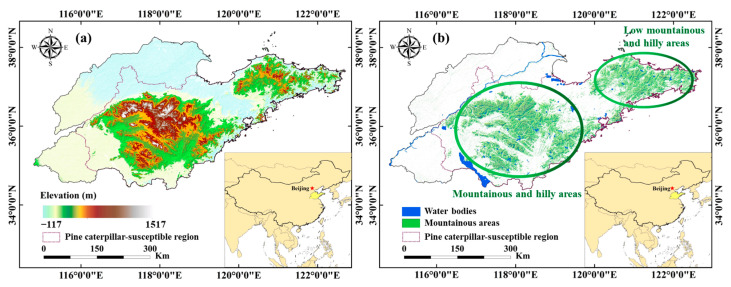
(**a**) Elevation and (**b**) mountainous areas of Shandong Province, China.

Shandong Province is located in the eastern coastal region of China, with geographical coordinates ranging from 34°22.9′ to 38°24.01′ N and 114°47.5′ to 122°42.3′ E (Figure 1a). The higher-elevation and mountainous areas are distributed in the central and eastern regions of Shandong Province (Figure 1b). Simultaneously, these regions are susceptible to pine caterpillar (*Dendrolimus* spp.) outbreaks, causing negative impacts on local forestry development and management [39]. The average annual temperature ranges from 11.2 to 14.4 °C.

### 2.2. Current Climate Data

The observed daily average temperature from 1979 to 2018 was collected from the National Tibetan Plateau/Third Pole Environment Data Center, with a spatial resolution of 0.1° × 0.1° (https://data.tpdc.ac.cn/, accessed on 19 February 2025) [58]. It was used to calculate the cumulative growing degree days for pine caterpillars, thereby obtaining the pine caterpillar voltinism. At the same time, it was the basis for assessing the accuracy of future climate data.

### 2.3. CMIP6 Future Climate Data

The CMIP6 provides global climate models (GCMs) under the Shared Socioeconomic Pathways (SSPs), which is an important foundational dataset for future climate change research [59]. First, the selected SSP scenarios are described in Table S1. The SSP1-2.6, SSP2-4.5, SSP3-7.0, and SSP5-8.5 scenarios represent low-, medium-, medium–high-, and high-radiative-forcing-path scenarios [60], respectively. This allows for a comprehensive analysis of the potential for future climate change and a comparative analysis of different scenarios. Second, the NEX-GDDP-CMIP6 high-resolution downscaled data used in this study was obtained from the NASA Center for Climate Simulation (https://www.nccs.nasa.gov, accessed on 19 February 2025) [60]. The bias correction and spatial decomposition method (BCSD) was employed to downscale the original GCM data to a spatial resolution of 0.25° × 0.25°. Among them, daily average temperatures were extracted from 21 GCMs (Table S2), including historical experimental data (1961–2014) and future climate scenario data (2015–2100). The following steps were taken to identify the optimal GCMs for simulating temperature in the study area:

- Multi-model ensemble (MME) [61]: Compared to using a single model, multi-model sets have better simulation performance and are widely used in climate change-related research. Specifically, it includes equal weight averaging and weighted averaging, which are used based on the performance of the model.
- Validation of GCM model performance [61]: The Taylor diagram can plot the correlation coefficient, root mean square error, and standard deviation between the simulated and the observed temperature on a polar coordinate diagram, allowing us to more intuitively identify better patterns. BCC-CSM2-MR, EC Earth3 Veg LR, and GFDL-ESM4 for equal weights were selected for better performance in simulating the daily average temperature in this study. The correlation coefficient was 0.93, the root mean square error was 0.42, and the standard deviation was 0.9 (Figure S1).

Finally, we output the future temperature for four different time periods (2021–2040, 2041–2060, 2061–2080, and 2081–2100, which will be referred to as the 2030s, 2050s, 2070s, and 2090s, respectively, throughout this paper) and compared it with the current period (1979–2018) as a baseline. All the data were uniformly interpolated onto a 0.05° × 0.05° grid to maintain consistency.

### 2.4. Modeling of Pine Caterpillar Voltinism

#### 2.4.1. Temperature-Based Indices and Threshold Definition

(1) Generation: the complete life cycle of an insect, from egg to adult.
(2) Voltinism: the number of generations an insect can produce in a year [19,22,42,62,63,64,65].
(3) Developmental zero (*T*_0_): the minimum temperature required for the development of an insect.
(4) Effective temperature accumulation (K): the effective thermal accumulation required for development from egg to adult.
(5) Cumulative growing degree day (CGDD): the sum of effective temperature accumulated over a year.

The developmental zero and effective temperature accumulation are two critical parameters for estimating voltinism using the growing degree day model [7,16]. This study referenced previous experimental research by Xia et al. [66], which employed a natural variable temperature method to measure the developmental parameters of pine caterpillars. Specifically, the experiments were carried out in outdoor (or forest-edge), iron-screen rearing chambers, with temperatures recorded using a thermohygrograph placed in a standard weather shelter. The developmental durations of larvae after overwintering, pupae, eggs, and larvae before overwintering were observed. Based on these experimental data, Xia et al. calculated the developmental zero and effective temperature accumulation for each life stage and the entire life cycle of pine caterpillars. The results are presented in Table 1. Similar research has been reported in [52,67].

#### 2.4.2. Estimation of Voltinism

Pine caterpillar voltinism is calculated as the ratio of the local cumulative growing degree day within a year to the effective thermal accumulation required for pine caterpillars to complete full generation. The *CGDD* is calculated as follows (Equation (1)):

(1) $$CGDD=\sum_{i=1}^{n}\left(T_{i}-T_{0}\right),\;T_{i}>T_{0}$$

where $T_{i}$ is the average temperature on day *i* in a calendar year (*n* = 1, 2, 3… 365), and $T_{0}=9.95\pm 0.61\;{}^{\circ}C$ (Table 1).

Voltinism is calculated as follows (Equation (2)):

(2) $$N\;=\frac{CGDD}{\mathrm{effective}\;\mathrm{temperature}\;\mathrm{of}\;\mathrm{pine}\;\mathrm{caterpillar}}$$

where N is the voltinism of the pine caterpillar and the effective temperature accumulation of the pine caterpillar = 1698.18 ± 48.18 degree days (Table 1).

Furthermore, the future changes in pine caterpillar voltinism are predicted, and the extent of such variations is quantified as Δ voltinism (which is equal to Voltinism_future period_ − Voltinism_current period_). The value > 0 denotes the increase in pine caterpillar voltinism, whereas a value < 0 denotes a decrease.

#### 2.4.3. Elevational and Latitudinal Gradients

Pine caterpillars are most likely to be found in low mountains and hilly regions below 500 m, occasionally at 500–1000 m, and less frequently at elevations above 1000 m [39]. In terms of population levels, the changes across various elevational and latitudinal gradients are of particular importance under climate change [22,68]. Therefore, the study area was partitioned into three elevational gradients (<500 m; 500–1000 m; and >1000 m) and five latitudinal gradients ((34.5–35° N]; (35–36° N]; (36–37° N]; (37–38° N]; and (38–38.5° N]) to analyze the future change patterns of voltinism along elevational and latitudinal gradients.

## 3. Results

### 3.1. CGDD Changes Under Current and Future Climate Scenarios

#### 3.1.1. Temporal Changes in CGDD

**Figure 2:**
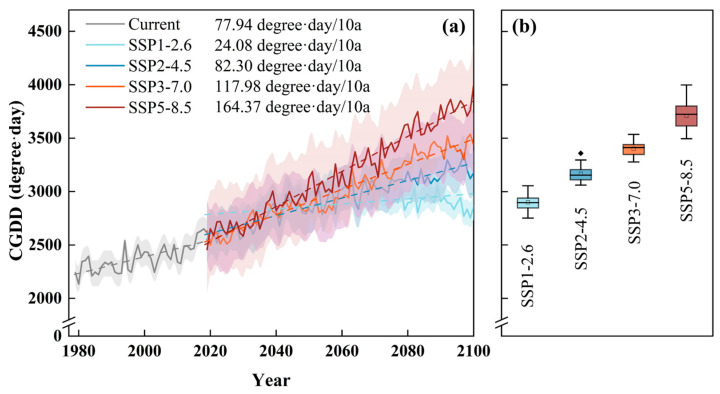
Temporal changes in the CGDD during the current period and future climatic scenarios. (**a**) The solid lines show the CGDD variations between 1979 and 2100, the dashed lines show linear trends of the CGDD, and the shaded areas show the 95% confidence interval. (**b**) The boxplot shows the CGDD values between 2081 and 2100. Note: /10a represents per decade. The rhomboid symbol represents outlier of the boxplot.

Currently, the CGDD for pine caterpillars in Shandong Province ranges from 2132.93 to 2651.25 degree days, with the minimum observed in 1980 and the maximum in 2017, showing an increasing trend of 77.94 degree days/10a (Figure 2a). This upward trend is expected to continue under future climate scenarios. According to Figure 2a,b, under the SSP1-2.6 scenario, CGDD is projected to increase at a rate of 24.08 degree days/10a, resulting in a rise of 523.45 degree days in the far future (2081–2100) compared to the current period. Under the SSP2-4.5, SSP3-7.0, and SSP5-8.5 scenarios, the increasing trends are projected to be 82.30, 117.98, and 163.37 degree days/10a, respectively. These increases correspond to 790.91 degree days, 1026.55 degree days, and 1336.43 degree days above current levels.

The fluctuations among different scenarios are relatively similar before the 2050s, but the differences are expected to widen thereafter. Notably, the SSP2-4.5 scenario is anticipated to align closely with the current warming trend. The SSP5-8.5 scenario shows the largest changes in CGDD, followed by SSP3-7.0, while the SSP1-2.6 scenario exhibits the smallest changes and fluctuations.

#### 3.1.2. Projected Geographic Patterns of CGDD

**Figure 3:**
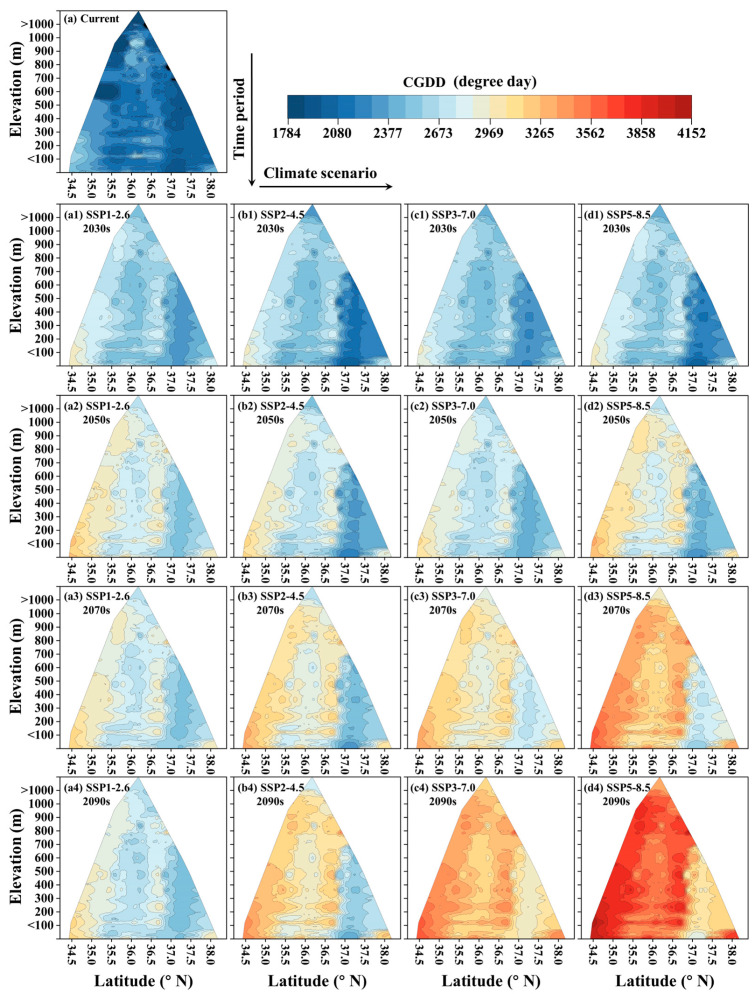
Elevational and latitudinal changes in CGDD under current period and future climatic scenarios. Time periods are displayed vertically and future climatic scenarios are displayed horizontally as follows: (**a**) current period and (**a1**–**a4**) SSP1-2.6, (**b1**–**b4**) SSP2-4.5, (**c1**–**c4**) SSP3-7.0, and (**d1**–**d4**) SSP5-8.5 scenarios. Color shifts from blue to red indicate a higher CGDD during each time period.

Figure 3 illustrates the CGDD changes over time (displayed vertically) and under different climate scenarios (displayed horizontally) along elevational and latitudinal gradients. Currently, the CGDD exhibits a decreasing trend from south to north in latitude and similarly decreases with increasing elevation (Figure 3a). Future CGDD values are projected to be higher than those of the current period. In the 2030s, the differences among the four climate scenarios are minimal, with similar change patterns expected (Figure 3(a1–d1)). However, after the 2050s, the differences between the scenarios become more pronounced. Overall, the variation in the CGDD is more substantial in mid–low-latitude (<36.5° N) and mid–low-elevation (<1000 m) regions compared to higher-latitude and -elevation regions (Figure 3(a2–d4)).

### 3.2. Pine Caterpillar Voltinism Changes Under Current and Future Climate Scenarios

#### 3.2.1. Temporal Changes in Pine Caterpillar Voltinism

**Figure 4:**
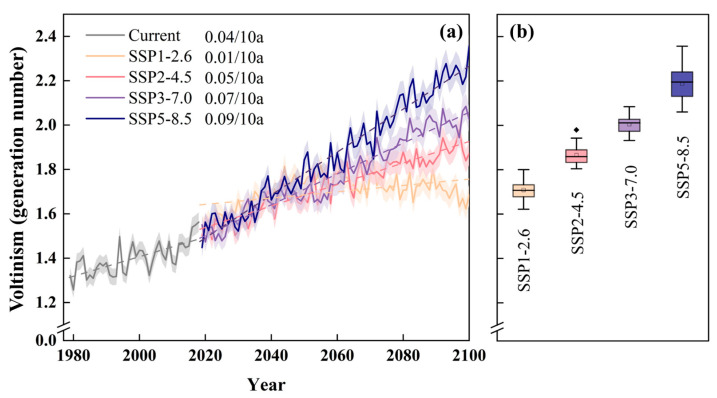
Temporal changes in pine caterpillar voltinism under current period and future climatic scenarios. (**a**) The solid lines show the variations in pine caterpillar voltinism between 1979 and 2100, the dashed lines show linear trends of pine caterpillar voltinism, and the shaded areas show the 95% confidence interval. (**b**) The boxplot shows the value of pine caterpillar voltinism between 2081 and 2100. Note: /10a represents per decade. The rhomboid symbol represents outlier of the boxplot.

The current voltinism of pine caterpillars in Shandong Province ranges from 1.26 to 1.56 generations (mean 1.40 ± 0.07), with the minimum and maximum values occurring in 1980 and 2018, respectively, showing an increasing trend at a rate of 0.04 generations/10a (Figure 4a). All four future climate scenarios are projected to show similar increasing patterns (Figure 4a). Under the SSP1-2.6 scenario, pine caterpillar voltinism is expected to increase at a rate of 0.01 generations/10a, reaching 1.71 ± 0.04 generations by the 2090s. In contrast, the SSP2-4.5, SSP3-7.0, and SSP5-8.5 scenarios predict increases of 0.05, 0.07, and 0.09 generations/10a, respectively, with voltinism reaching 1.87 ± 0.04, 2.00 ± 0.04, and 2.19 ± 0.07 generations by the 2090s (Figure 4b).

Furthermore, the difference between the scenarios is relatively small before the 2050s, after which it is expected to increase. Notably, SSP2-4.5 is expected to be the climate scenario that is closer to the trend in pine caterpillar voltinism over the current period. The SSP5-8.5 scenario is projected to have the greatest variation in pine caterpillar voltinism, followed by SSP3-7.0, whereas the SSP1-2.6 scenario is expected to have the smallest variations and fluctuations.

#### 3.2.2. Projected Geographic Patterns of Pine Caterpillar Voltinism

**Figure 5:**
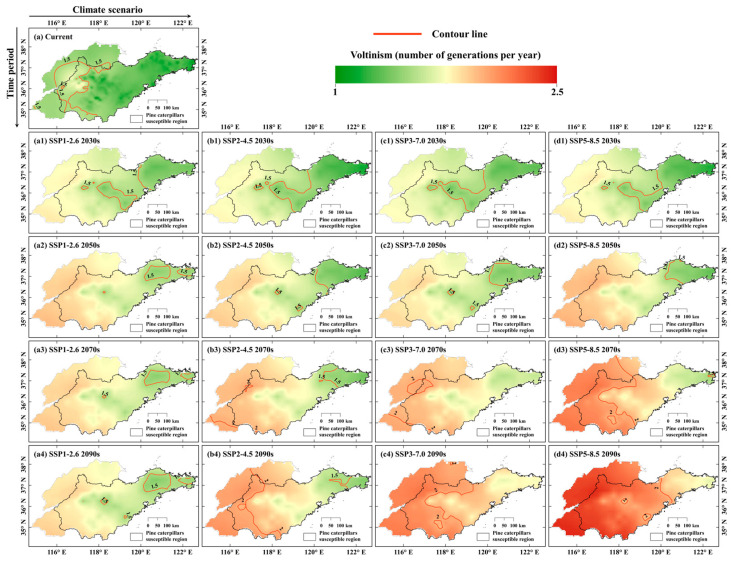
Projected geographic patterns of pine caterpillar voltinism under current period and future climatic scenarios. Time periods are displayed vertically and future climatic scenarios are displayed horizontally as follows: (**a**) current period and (**a1**–**a4**) SSP1-2.6, (**b1**–**b4**) SSP2-4.5, (**c1**–**c4**) SSP3-7.0, and (**d1**–**d4**) SSP5-8.5 scenarios. Color shifts going from green to red indicate higher voltinism during each time period.

Figure 5 shows the changes in pine caterpillar voltinism over time (displayed vertically) and under different climate scenarios (displayed horizontally). In the current period, pine caterpillar voltinism is highest in the western region at about 1.5 generations/year, in the central mountainous region at about 1.2–1.3 generations/year, and in the eastern low mountains and hilly region at about 1.1–1.2 generations/year (Figure 5a). The model predicts a similar geographic pattern of pine caterpillar voltinism in the future, decreasing from west to east. Specifically, in the 2030s, pine caterpillar voltinism is more similar under the four climate scenarios, increasing to approximately 1.7–1.8 generations/year in the western region, 1.5–1.6 generations/year in the central mountainous region, and 1.3–1.4 generations/year in the eastern low mountains and hilly region (Figure 5(a1–d1)). In the 2050s, the predicted results show that they are within 2 generations/year in all regions (Figure 5(a2–d1)). After the 2070s, pine caterpillar voltinism appears to be more than 2 generations/year in some areas, such as the southwestern region in the SSP2-4.5 scenario (Figure 5(b3)).

For the climate scenarios, as radiative forcing rises, the area of pine caterpillar voltinism greater than 2 generations/year is more widespread. For example, in the SSP5-8.5 scenario during the 2090s (Figure 5(d4)), it increases to about 2.3–2.4 generations/year in the western region, 2–2.2 generations/year in the central mountainous region, and 1.7–1.9 generations/year in the eastern low mountains and hilly region, which is an increase of about 0.8 generations compared to the current period. In general, there are more generations at lower elevations and in warmer areas at lower latitudes.

### 3.3. Predicted Elevational Patterns of Pine Caterpillar Voltinism Under Future Climate Scenarios

#### 3.3.1. Projected Increase in Pine Caterpillar Voltinism Across Elevational Gradients

**Figure 6:**
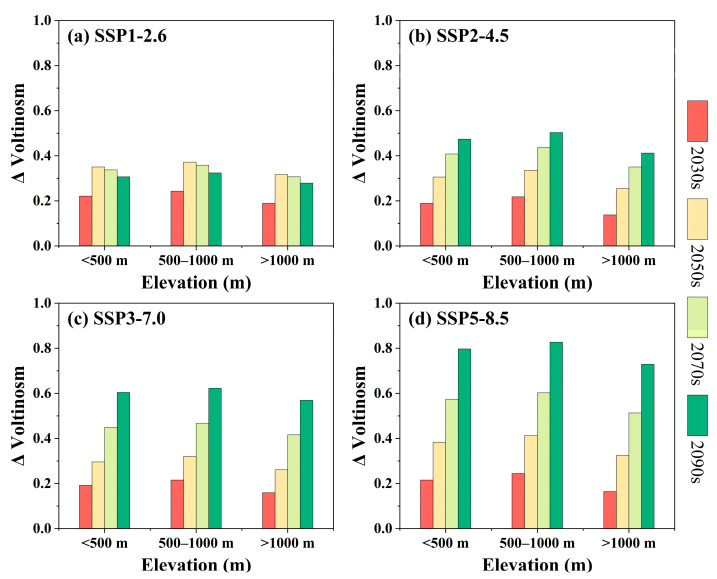
Elevational patterns of pine caterpillar voltinism under future climatic scenarios. (**a**) SSP1-2.6, (**b**) SSP2-4.5, (**c**) SSP3-7.0, and (**d**) SSP5-8.5 scenarios.

Figure 6 reveals the changes pattern in pine caterpillar voltinism along the elevation gradient under future climate scenarios. In general, the model predicts an increase in pine caterpillar voltinism across all elevation gradients, with this increase being more pronounced under the SSP5-8.5 scenario (Figure 6d). In terms of magnitude, the greatest increase in pine caterpillar voltinism is projected at 500–1000 m, followed by 500 m, with the smallest at >1000 m. This indicates that the increase in pine caterpillar voltinism is more pronounced at elevations <1000 m. However, the specific increases vary depending on the time period and climate scenario. For example, during the same period (for the 2090s), the pine caterpillar voltinism at 500–1000 m increases by 0.32, 0.50, 0.62, and 0.83 generations under the SSP1-2.6, SSP2-4.5, SSP3-7.0, and SSP5-8.5 scenarios, respectively (Figure 6). Similarly, under the same scenario (for the SSP5-8.5 scenario), the pine caterpillar voltinism at 500–1000 m increases by 0.24, 0.41, 0.60, and 0.83 generations in the 2030s, 2050s, 2070s, and 2090s, respectively (Figure 6d).

#### 3.3.2. Projected Change in Pine Caterpillar Voltinism with Temperature Increase Across Elevational Gradients

**Figure 7:**
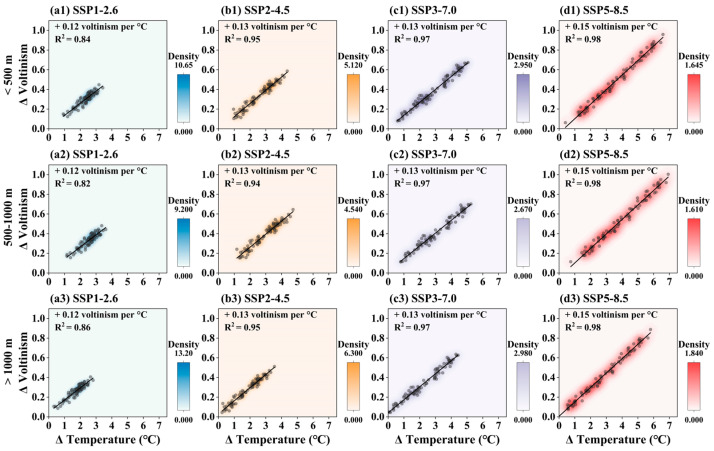
The relationship between pine caterpillar voltinism and temperature along elevational gradients under future climatic scenarios. The y-axis is the change extent in voltinism and the x-axis is the change extent in average annual temperature. Scatter plots show the value of pine caterpillar voltinism, and solid lines show the fitting line of pine caterpillar voltinism (n = 82). (**a1**–**a3**) SSP1-2.6, (**b1**–**b3**) SSP2-4.5, (**c1**–**c3**) SSP3-7.0, and (**d1**–**d3**) SSP5-8.5 scenarios.

The relationship between pine caterpillar voltinism and changes in average annual temperature along elevational gradients is shown in Figure 7. Voltinism increases consistently with rising temperatures across all elevation gradients and climate scenarios. The trend in pine caterpillar voltinism across different elevations shows an increase of 0.12 to 0.15 generations per 1 °C rise in average annual temperature, explaining over 82% of the variation in voltinism. When the average annual temperature in Shandong Province increases by 1.5 °C or 2 °C, all climate scenarios exhibit similar patterns. With a 1.5 °C increase, voltinism is projected to increase by approximately 0.2 generations at elevations <500 m, 0.21 generations at 500–1000 m, and 0.23 generations at elevations >1000 m. With a 2 °C increase, voltinism is projected to rise by approximately 0.24 generations at elevations <500 m, 0.26 generations at 500–1000 m, and 0.3 generations at >1000 m. Overall, these projections are consistent across all climate models, although some uncertainty remains regarding regional warming patterns.

### 3.4. Predicted Latitudinal Patterns of Pine Caterpillar Voltinism Under Future Climate Scenarios

#### 3.4.1. Projected Increase in Pine Caterpillar Voltinism Across Latitudinal Gradients

**Figure 8:**
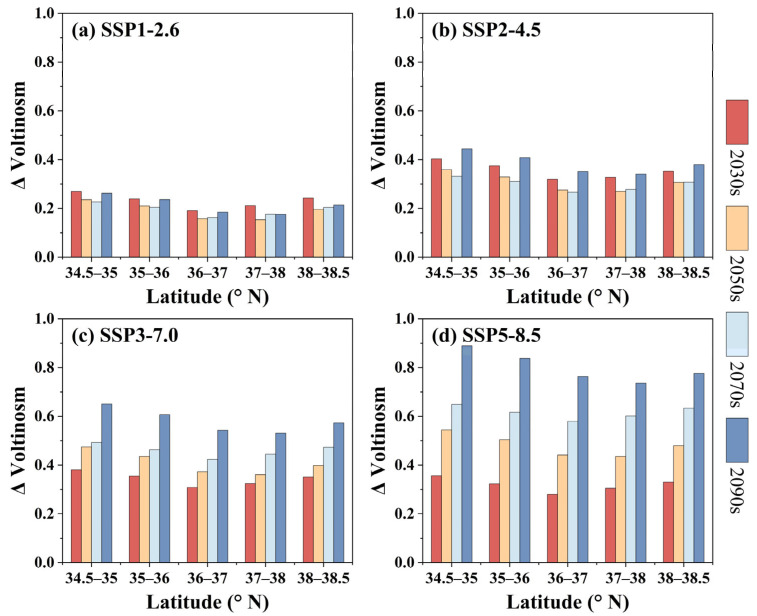
Latitudinal changes pattern in pine caterpillar voltinism under future climatic scenarios. (**a**) SSP1-2.6, (**b**) SSP2-4.5, (**c**) SSP3-7.0, and (**d**) SSP5-8.5 scenarios.

Figure 8 reveals the change pattern in pine caterpillar voltinism along the latitude gradient under future climate scenarios. In general, the model predicts an increase in pine caterpillar voltinism across all latitudinal gradients, with this increase being more pronounced under the SSP5-8.5 scenario (Figure 8d). In terms of magnitude, the greatest increase in pine caterpillar voltinism is projected at 34.5–35° N, followed by 35–36° N and 38–38.5° N. This indicates that the increase in pine caterpillar voltinism is more pronounced at lower latitudes (approximately <36.5° N in this study). However, the specific increases vary depending on the time period and climate scenario. For example, during the same period (for the 2090s), the pine caterpillar voltinism at 35–36° N increases by 0.24, 0.41, 0.61, and 0.83 generations under the SSP1-2.6, SSP2-4.5, SSP3-7.0, and SSP5-8.5 scenarios, respectively (Figure 8). Similarly, under the same scenario (for the SSP5-8.5 scenario), the pine caterpillar voltinism at 35–36° N increases by 0.32, 0.41, 0.62, and 0.83 generations in the 2030s, 2050s, 2070s, and 2090s, respectively (Figure 8).

#### 3.4.2. Projected Change in Pine Caterpillar Voltinism with Temperature Increase Across Latitudinal Gradients

**Figure 9:**
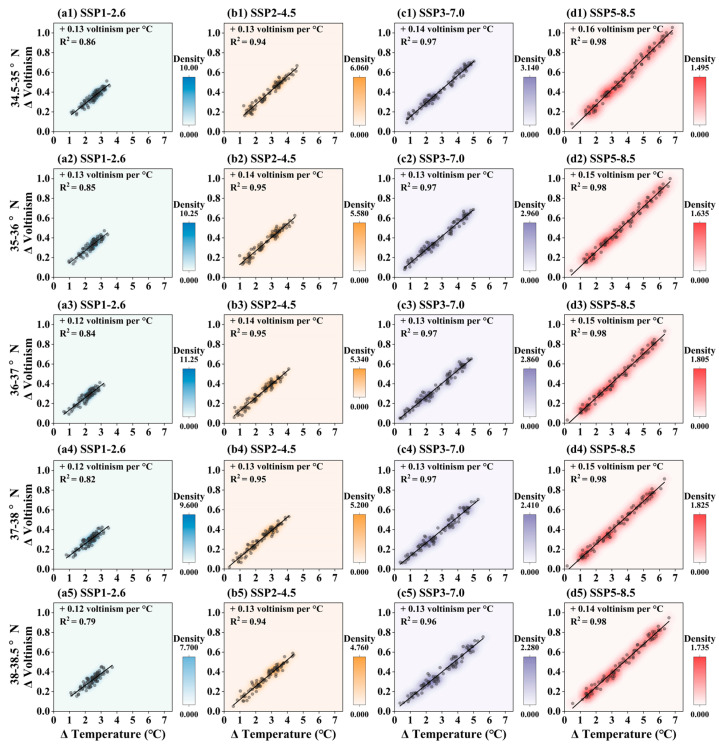
Relationship between pine caterpillar voltinism and temperature along latitudinal gradients under future climatic scenarios. The y-axis is the change extent in voltinism and the x-axis is the change extent in average annual temperature. Scatter plots show the value of pine caterpillar voltinism, and solid lines show the fitting line of pine caterpillar voltinism (n = 82). (**a1**–**a5**) SSP1-2.6, (**b1**–**b5**) SSP2-4.5, (**c1**–**c5**) SSP3-7.0, and (**d1**–**d5**) SSP5-8.5 scenarios.

The relationship between pine caterpillar voltinism and changes in average annual temperature along latitudinal gradients is shown in Figure 9. Voltinism increases consistently with rising temperatures across all latitude gradients and climate scenarios. The trend in pine caterpillar voltinism across different elevations shows an increase of 0.12 to 0.16 generations per 1 °C rise in average annual temperature, explaining over 79% of the variation in voltinism. When the average annual temperature in Shandong Province increases by 1.5 °C or 2 °C, all climate scenarios exhibit similar patterns. With a 1.5 °C increase, voltinism is projected to increase by approximately 0.21 generations at 34.5–35° N, 0.20 generations at 35–36° N, 0.19 generations at 36–37° N, 0.18 generations at 37–38° N, and 0.17 generations at 38–38.5° N. With a 2 °C increase, voltinism is projected to rise by approximately 0.28 generations at 34.5–35° N, 0.27 generations at 35–36° N, 0.26 generations at 36–37° N, 0.25 generations at 37–38° N, and 0.24 generations at 38–38.5° N. Overall, these projections are consistent across all climate models, although some uncertainty remains regarding regional warming patterns.

## 4. Discussion

The degree day model demonstrates an increase in pine caterpillar voltinism due to rising temperature in this study. Previous experimental studies documented one generation per year for the pine caterpillar in Shandong Province [44]. Our results show that the average voltinism ranged between 1.26 and 1.56 generations during 1979–2018 and is projected to increase to 1.71 ± 0.04 (SSP1-2.6), 1.87 ± 0.04 (SSP2-4.5), 2.00 ± 0.04 (SSP3-7.0), and 2.19 ± 0.07 generations (SSP5-8.5) per year by the 2090s. Choi et al. (2011) observed a shift from univoltine to bivoltine species in central Korea during 1999–2000 [52]. However, our temporal framework facilitates the long-term monitoring of climate change and predictions of pine caterpillar voltinism. A functional explanation for the increase in voltinism could be the earlier onset of the flight period, which extends the season and allows for the production of additional generations [17,69]. Consistent with other species, the onset of pine caterpillar emergence has shifted to earlier dates [70]. Another explanation is that rising temperature accelerates larval growth and development [18], as seen in European butterflies [17]. Warmer temperature can benefit some insect populations by accelerating growth during the growing season, thereby enabling more generations. This is particularly true for multivoltine species, where warming allows an increase in generations, while univoltine species are less affected [71]. Conversely, species in tropical and subtropical regions, which already experience high temperature for most of the year, are more likely to encounter temperatures that exceed their upper thermal limits, limiting further increases in voltinism [72]. This is because warmer latitudes are already close to optimal temperatures for insect activity, and further warming may not promote population growth effectively, potentially limiting population size due to physiological tolerance limits [24]. On the other hand, warming in colder latitudes will provide more favorable conditions for insect activity, boosting population growth potential. In our study area, the average temperature is projected to increase by 2.37 °C (SSP1-2.6), 3.45 °C (SSP2-4.5), 4.35 °C (SSP3-7.0), and 5.49 °C (SSP5-8.5) by the 2090s, compared to the current temperature [70]. The projected temperature increase varies by geographic location, with the western plains expected to experience the greatest warming, followed by the mountainous and hilly regions in the central and southern areas and the low mountains and hilly regions in the east. By the 2090s, under the SSP5-8.5 scenario, the estimated average temperature increase will result in a shift in pine caterpillar voltinism, with western locations showing an increase from 1.5 generations to 2.3–2.4 generations per year, central mountainous areas shifting from 1.2 to 1.3 to 2–2.2 generations per year, and eastern low mountain and hilly regions increasing from 1.1 to 1.2 to 1.7–1.9 generations per year. These projections align with the findings of Bentz et al. (2023) [22]. Our study emphasizes the different temporal patterns of voltinism changes, highlighting the importance of localized climate change modeling and the management of regional-scale variability [73].

Notably, studies have shown that latitude and elevation significantly affect insect life cycles [9,68,74,75,76], especially in northern latitudes where the growing season is shorter [9,32]. In our elevation gradient predictions, the most pronounced changes in pine caterpillar voltinism occur at 500–1000 m, followed by elevations <500 m. This suggests that regions below 1000 m experience a greater impact. This is likely due to the natural decrease in average temperature with increasing elevation. Jacques et al. (2019) observed frequent below-freezing temperatures and frosts in higher-elevation areas within the study region [24]. Overall, the lower temperatures at elevations >1000 m in Shandong Province explain the fewer generations predicted in these areas. In the latitude gradient predictions, changes in voltinism are more pronounced in the low- and high-latitude regions (34.5–36° N and 38–38.5° N), while the mid-latitude regions (36–38° N) exhibit smaller changes. Typically, voltinism decreases with latitude in species capable of multiple generations [19,77,78]. However, Bentz et al. (2014) demonstrated that mountain pine beetle populations evolving in cooler northern latitudes develop faster than those at warmer low-latitude regions [68]. As temperatures rise, the transition from univoltine to multivoltine species is shifting toward higher latitudes [78]. Latitudinal changes in insect voltinism are driving alterations in various life-history traits, including growth, development, and the ability to cope with winter conditions [79]. Another possible reason for the lower voltinism in the 36–38° N region of Shandong Province is the mountainous and high-elevation distribution, where cooler temperatures lead to fewer generations. The specific changes in voltinism also depend on the sensitivity of each elevation and latitude gradient to rising temperature [22,23,24].

This study provides long-term projections, offering insights into potential trends under climate change. All four climate scenarios predict an increase in pine caterpillar voltinism, with more pronounced increases under high-emission scenarios. Similar findings have been reported by Jacques et al. (2019) [24] and Santos et al. (2021) [26]. A small portion of the changes in voltinism can be explained by the climate change scenarios [18,24]. This result is reasonable, as global warming is a temporally connected process [80]. Despite the difference of up to 3.12 °C in the expected temperature increase between the SSP1-2.6 and SSP5-8.5 scenarios by the 2090s, the estimated changes in voltinism are similar in both scenarios. In fact, these patterns are closely linked to climate change and the extent of warming. Tobin et al. (2008) illustrated that increases in average temperatures greater than 2 °C can noticeably affect insect voltinism [20]. Our results show that pine caterpillar voltinism is projected to increase by approximately 0.15 generations, 0.2 generations, and 0.28 generations per year with increases of 1 °C, 1.5 °C, and 2 °C in the average annual temperature, respectively. Overall, voltinism increases with temperature [28], and we speculate on the possible changes.

Existing research indicates that the direct impact of temperature may be greater than any other factor, and it has been identified as the primary abiotic factor directly affecting herbivorous insects [9,76,81,82]. Our predictions are based on daily average temperature, as it allows for long-term forecasts of voltinism on a regional scale under future climatic scenarios. However, photoperiod also plays a crucial role in inducing diapause, and other factors such as insect emergence timing, host plant availability, precipitation, humidity, and predation also have significant influences [83]. The increase in extreme weather events is one of the predicted consequences of climate change, and it is also a relevant issue that needs to be considered [27,84]. As a result, there are some limitations to using the growing degree day method in this study. Additionally, insects will exhibit adaptation to climate warming [6]. The degree day constants and developmental thresholds of the pine caterpillar may shift with changing climatic conditions, leading to an increased ability to tolerate higher temperature. This study used a developmental threshold temperature of 9.95 ± 0.61 °C for the pine caterpillar, which introduced a certain degree of bias in long-term voltinism predictions, an unavoidable limitation in projection studies. According to the temperature limits observed in congeneric species [46], the development and survival of the pine caterpillar in Shandong Province are expected to benefit from rising temperature. The model used in this study is likely more applicable to multivoltine insect species at mid-to-high latitudes or in temperate regions where future temperature changes remain within the optimal range for insect development. The length of the growing season is another potential environmental limiting factor for insect development [85]. Future research should investigate how climate change affects the life history of insects, including developmental periods in each life stage and their interactions with host species.

## 5. Conclusions

This study demonstrates that the cumulative growing degree day (CGDD) is projected to increase by 523.45, 790.91, 1026.55, and 1336.43 degree days by the end of this century under the SSP1-2.6, SSP2-4.5, SSP3-7.0, and SSP5-8.5 scenarios, respectively. This suggests an increase in pine caterpillar voltinism in a warming climate, with voltinism expected to rise from 1.40 ± 0.07 (current period) to 1.71 ± 0.04, 1.87 ± 0.04, 2.0 ± 0.04, and 2.19 ± 0.07 generations under the SSP1-2.6, SSP2-4.5, SSP3-7.0, and SSP5-8.5 scenarios, respectively. Model predictions indicate the greatest increase in the western plain, followed by the central and eastern regions of Shandong Province. From the perspective of elevation and latitude gradients, the lower-elevation (<1000 m) and lower-latitude (34–34.5° N) regions are expected to experience more generations in the future. The specific magnitude of these changes varies by time period and climate scenarios. Overall, pine caterpillar voltinism will increase with climate warming, with projections showing an increase of approximately 0.15, 0.2, and 0.28 generations per year for every 1 °C, 1.5 °C, and 2 °C rise in the average annual temperature, respectively.

## Figures and Tables

**Table 1:** The developmental zero and effective temperature accumulation for pine caterpillars (*Dendrolimus spectabilis* Butler) in each stage and after complete generation [66].

Developmental Stage	Larvae After Overwintering	Pupae	Egg	Larvae Before Overwintering	Complete Generation
Developmental zero (°C)	9.72 ± 0.62	13.38 ± 0.35	9.62 ± 0.29	8.94 ± 0.77	9.95 ± 0.61
Effective temperature accumulation (degree days)	757.92 ± 55.34	234.27 ± 7.98	137.16 ± 2.78	568.84 ± 66.15	1698.18 ± 48.18

## Data Availability

The daily average temperature was downloaded from the National Tibetan Plateau/Third Pole Environment Data Center (https://data.tpdc.ac.cn/, accessed on 19 February 2025) [58]. The Coupled Model Comparison Project Phase 6 (CMIP6) model data are freely available from the website https://www.nccs.nasa.gov/, accessed on 19 February 2025 [60].

## Supplementary Materials

The following supporting information can be downloaded at https://www.mdpi.com/article/10.3390/insects16030249/s1. Table S1: A description of future climate scenarios used in this study; Table S2: A list of 21 models selected for this study; Figure S1: The Taylor diagram for GCMs in this study. The position of the model being close to the observation indicates better performance.
