# Supplementary material for: Climate Warming Increases the Voltinism of Pine Caterpillar (Dendrolimus spectabilis Butler): Model Predictions Across Elevations and Latitudes in Shandong Province, China
Source: Insects. 2025 Feb 28;16(3):249. doi: 10.3390/insects16030249 (PMC11943339; doi:10.3390/insects16030249)
Supplement: Supplementary file 1 [file insects-16-00249-s001.zip › insects-3468039-supplementary.docx]

**Table S1.** Description of future climate scenarios used in this study.

| **Scenario** | **Radiative Forcing Types** | **SSPs (Socio-Economic Pathway)** | **Radiative Forcing by the Year 2100 (W·m^−2^)** | **Description** |
| --- | --- | --- | --- | --- |
| SSP1-2.6 | Low | 1 sustainable development | 2.6 | The combined effects of low vulnerability, low mitigation pressure and low radiation forcing. The global average temperature will rise by about 1.8 °C by the year 2100. |
| SSP2-4.5 | Medium | 2 Moderate development | 4.5 | The combination of moderate social vulnerability and moderate radiation forcing. The global average temperature will rise by about 2.7 °C by the year 2100. |
| SSP3-7.0 | High | 3 Local development | 7.0 | The combination of high social vulnerability and relatively high radiation forcing. The global average temperature will rise by about 4 °C by the year 2100. |
| SSP5-8.5 | High | 5 Conventional development | 8.5 | The highest emission of all scenario predictions. The global average temperature will rise by about 4.4 °C by the year 2100. |

**Table S2.** List of 21 models selected for this study.

| **No.** | **CMIP6 models** | **Institution** | **Resolution** |
| --- | --- | --- | --- |
| 1 | ACCESS-CM2 | Commonwealth Scientific and Industrial Research Organization, Australia | 1.875° × 1.25° |
| 2 | ACCESS-ESM1-5 | Commonwealth Scientific and Industrial Research Organization, Australia | 1.875° × 1.24° |
| 3 | BCC-CSM2-MR | Beijing Climate Center，China | 1.125° × 1.125° |
| 4 | CanESM5 | Canadian Centre for Climate Modelling and Analysis, Canada | 2.8125° × 2.8125° |
| 5 | CMCC-ESM2 | Fondazione Centro Euro-Mediterraneo sui Cambiamenti Climatici, Italy | 1.25° × 0.9375° |
| 6 | CNRM-CM6-1 | Centre National de Recherches Météorologiques (CNRM), France | 1.4° × 1.4° |
| 7 | EC-Earth3 | EC-EARTH consortium, European | 0.703° × 0.703° |
| 8 | EC-Earth3-CC-Veg-LR | EC-EARTH consortium, European | 1.125° × 1.125° |
| 9 | FGOALS-g3 | Chinese Academy of Sciences, China | 2.0° × 2.0° |
| 10 | GFDL-CM4 | NOAA Geophysical Fluid Dynamics Laboratory, USA | 1.25° × 1° |
| 11 | GFDL-ESM4 | NOAA Geophysical Fluid Dynamics Laboratory, USA | 1.25° × 1° |
| 12 | GISS-E2-1-G | NASA-GISS | 2.5° × 2.0° |
| 13 | INM-CM4-8 | Institute for Numerical Mathematics, Russian Academy of Science, Russia | 2.0° × 1.5° |
| 14 | INM-CM5-0 | Institute for Numerical Mathematics, Russian Academy of Science, Russia | 2.0° × 1.5° |
| 15 | IPSL-CM6A-LR | Institute Pierre Simon Laplace (IPSL), France | 2.5° × 1.26° |
| 16 | MIROC6 | National Institute for Environmental Studies, Ibaraki, Japan | 1.4° × 1.4° |
| 17 | MPI-ESM1-2-HR | Max Planck Institute for Meteorology, Germany | 0.9375° × 0.9376° |
| 18 | MPI-ESM1-2-LR | Max Planck Institute for Meteorology, Germany | 1.875° × 1.875° |
| 19 | MRI-ESM2-0 | Meteorological Research Institute, Tsukuba, Japan | 1.125° × 1.125° |
| 20 | NESM3 | Nanjing University of Information Science and Technology, China | 1.875° × 1.875° |
| 21 | NorESM2-MM | Norwegian Climate Centre (NorCC), Norway | 1.25° × 0.94° |


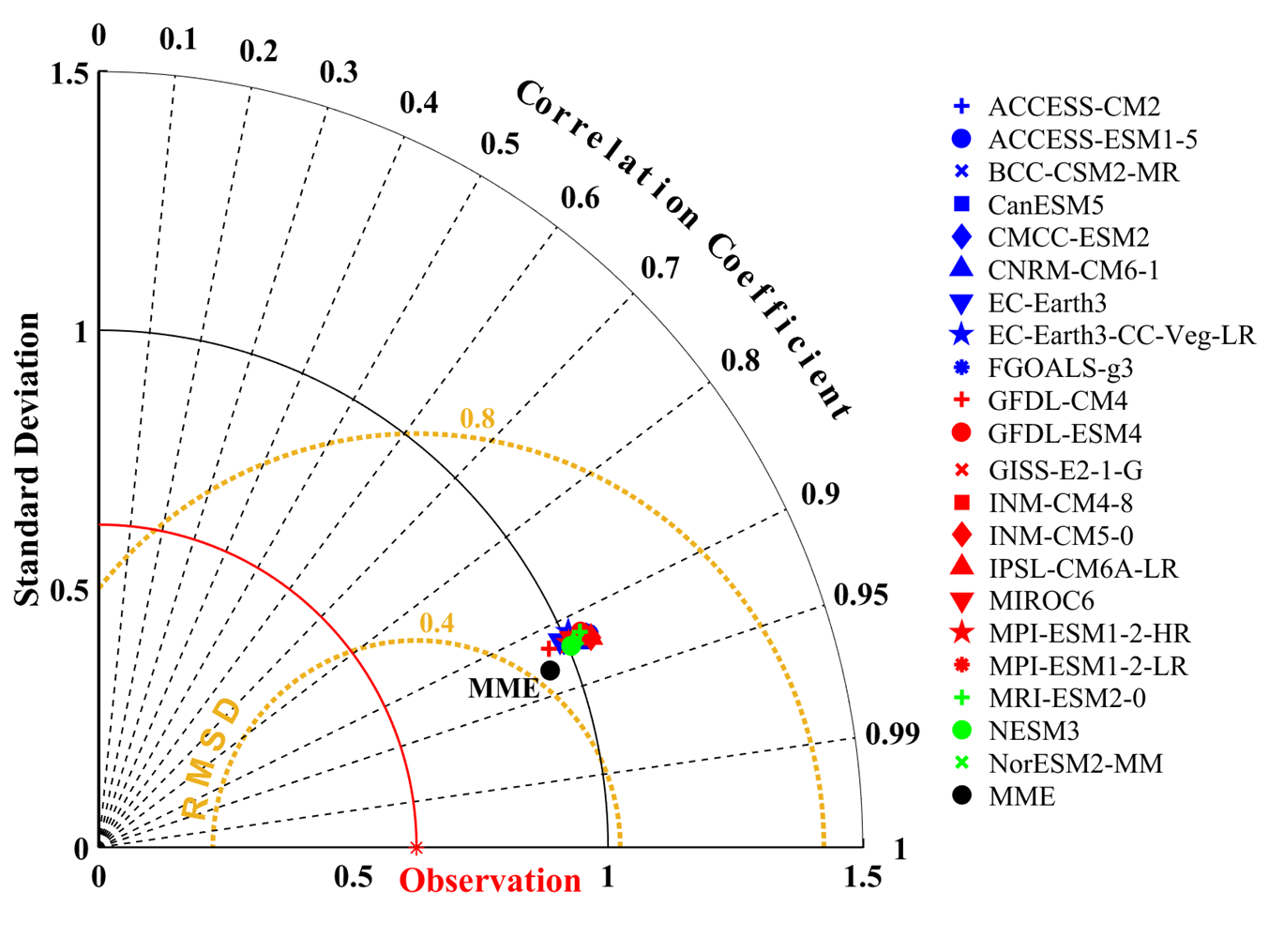


**Figure S1.** The Taylor diagram for GCMs in this study. The position of the model close to the observation indicates better performance.
